# Supplementary material for: The shared genetic architecture and evolution of human language and musical rhythm
Source: Nat Hum Behav. 2024 Nov 21;9(2):376–90. doi: 10.1038/s41562-024-02051-y (PMC11860242; doi:10.1038/s41562-024-02051-y)
Supplement: Supplementary file 1 — Supplementary Figs. 1–9. [file 41562_2024_2051_MOESM1_ESM.pdf]

nature human behaviour

Supplementary information https://doi.org/10.1038/s41562-024-02051-y

The shared genetic architecture and evolution of human language and musical rhythm

In the format provided by the authors and unedited

Supplementary Information – Table-of-contents

Supplementary Figures

Figure S1. Genomic SEM model diagrams for common pathway and independent pathways models.

Figure S2. The Quantile-Quantile (QQ) plots of F_{gRI-D} mvGWAS prior to and after Genomic Control (GC) correction.

Figure S3. Manhattan plots for multivariate GWASs of dyslexia and musical rhythm obtained using Genomic SEM common pathway model, N-weighted GWAMA and CPASSOC.

Figure S4. Manhattan plots for the independent genetic factors of dyslexia and musical rhythm impairment obtained using Genomic SEM independent pathways model.

Figure S5. Genetic correlation analysis results between 88 traits that are significantly correlated (P_{FDR}<0.05) either with dyslexia or musical rhythm. Traits were curated from Doust et al. (2022) and Niarchou et al. (2022).

Figure S6. A dendrogram showing the hierarchical clustering of highly genetically correlated (|rg| >0.80) traits, and a knee-point algorithm showing seven representative clusters.

Figure S7. Genetic correlation analysis results between 49 selected traits and dyslexia and musical rhythm impairment independent factors and F_{gRI-D}.

Figure S8. Brain plots showing five left hemisphere white-matter tracts investigated in our local genetic correlation analysis with F_{gRI-D}.

Figure S9. LocusZoom plot of the chr20: 30,569,660-32,484,506 locus, the region which we identified using local genetic correlation analysis between F_{gRI-D} and our left hemisphere Superior Longitudinal Fasciculus-I white-matter tract connectivity GWAS.

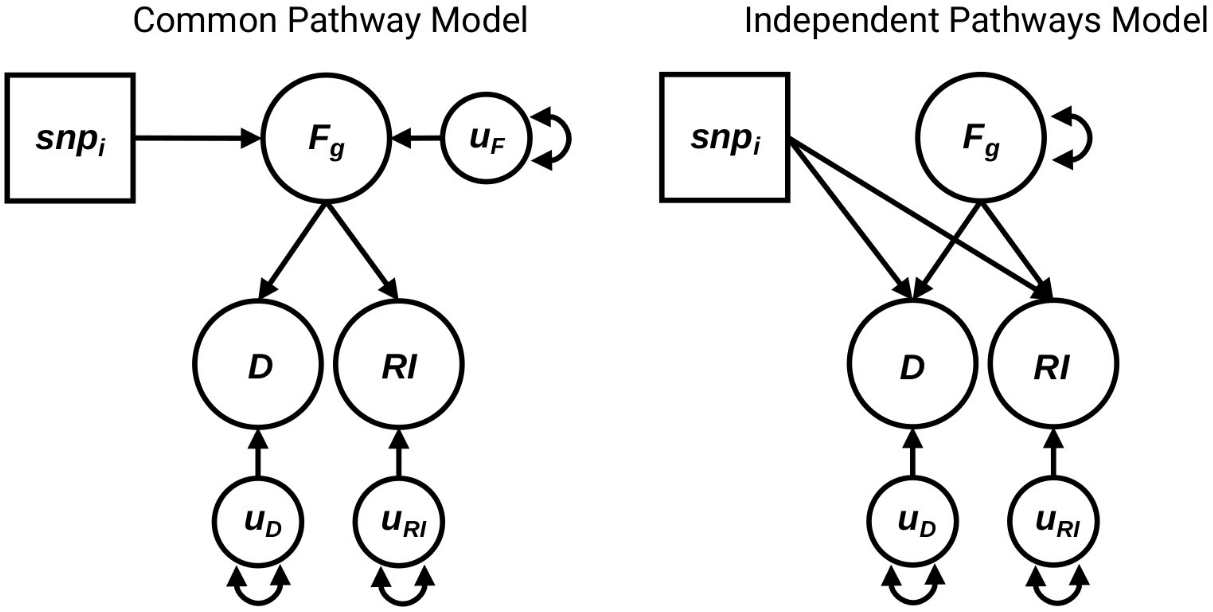

Supplementary Figure 1: Genomic SEM model diagrams for common pathway (left) and independent pathways (right) models. The models were used to perform a bivariate GWAS using Genomic SEM, and for the identification of common and independent genetic factors. D: dyslexia, RI: rhythm impairment, F_g: Common factor, u_D: residual variance of dyslexia, u_{RI}: residual variance of musical rhythm impairment, u_F: residual variance of the common factor, snp_i: i^{th} SNP regression.

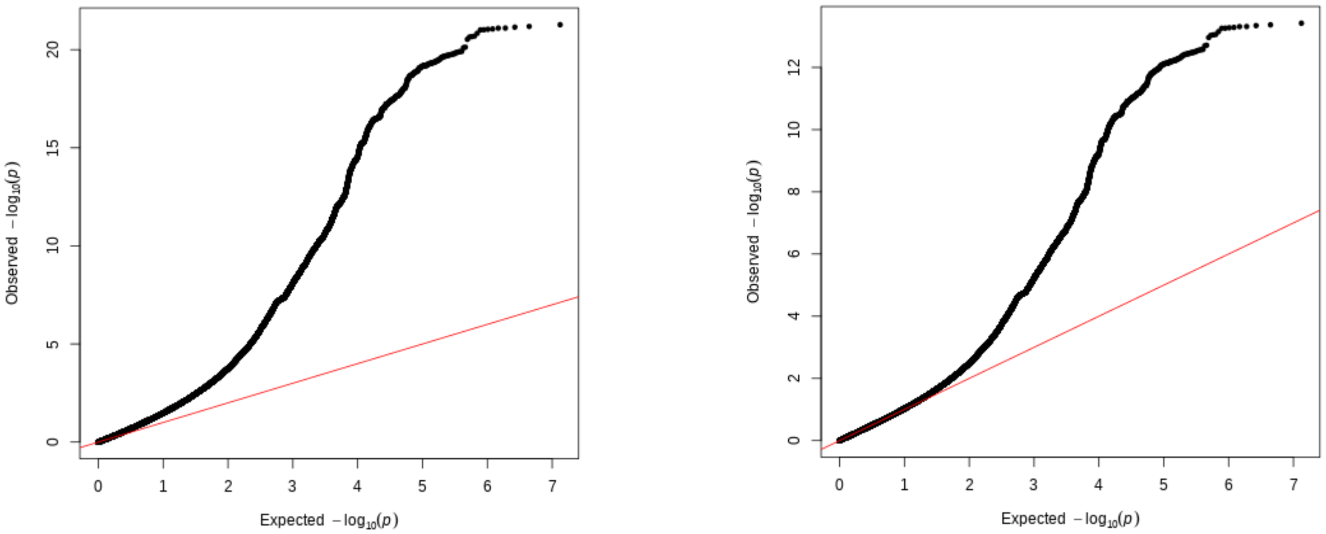

Supplementary Figure 2: The Quantile-Quantile (QQ) plots of $F_{gRI\text{-}D}$ mvGWAS show observed (*y* axis) and expected (*x* axis) -$\log_{10}$(P) values *prior to* (left) and *after* (right) GC correction.

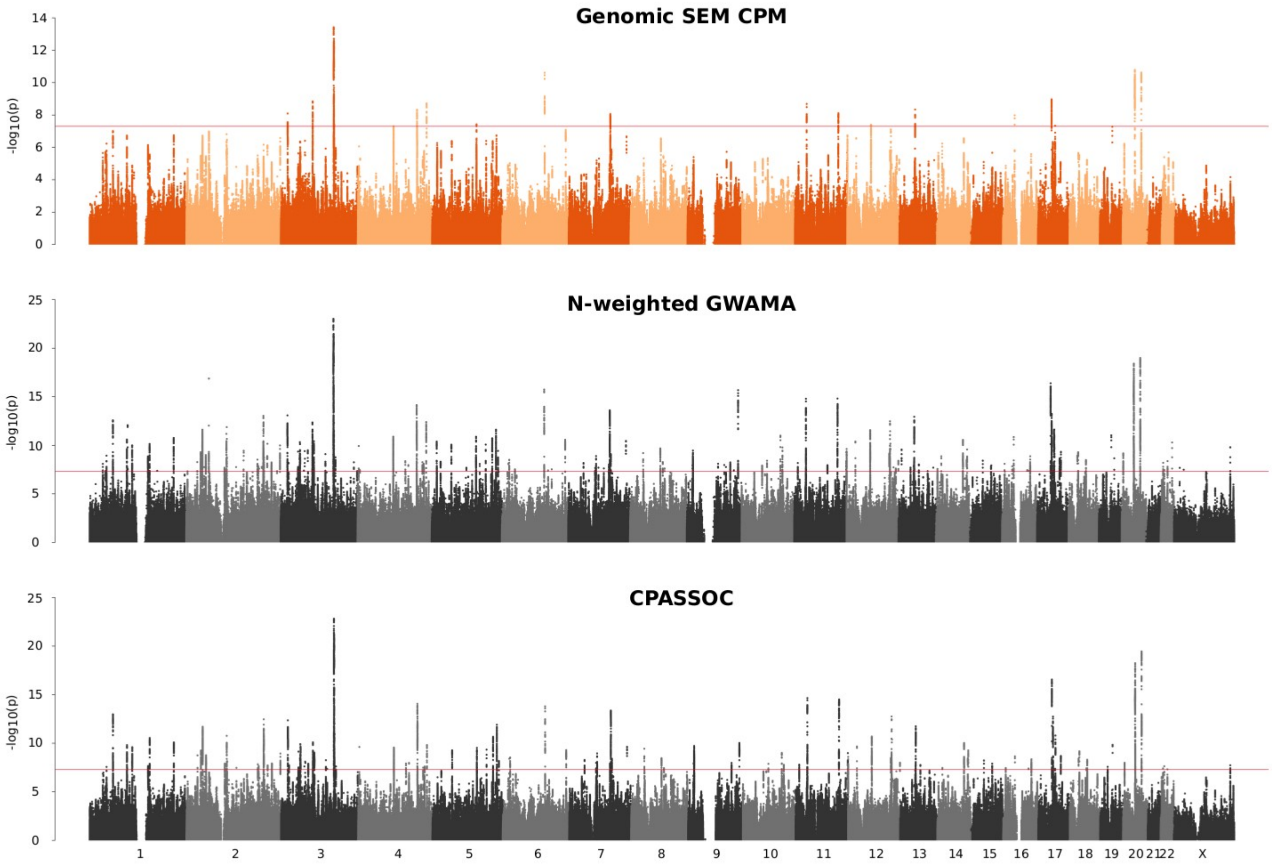

Supplementary Figure 3: Manhattan plots for Genomic SEM CPM ($F_{gRI\text{-}D}$), N-weighted GWAMA, and CPASSOC (see *Multivariate genome-wide association studies* in the Methods for details of each method). Each data point represents a SNP. The *y* axes show -log10(P) values, and the *x* axes show genomic coordinates. The red lines correspond to the genome-wide significance threshold ($P<5\times10^{-8}$). $N_{rhythm\ impairment}$=606,825, $N_{dyslexia}$=1,138,870.

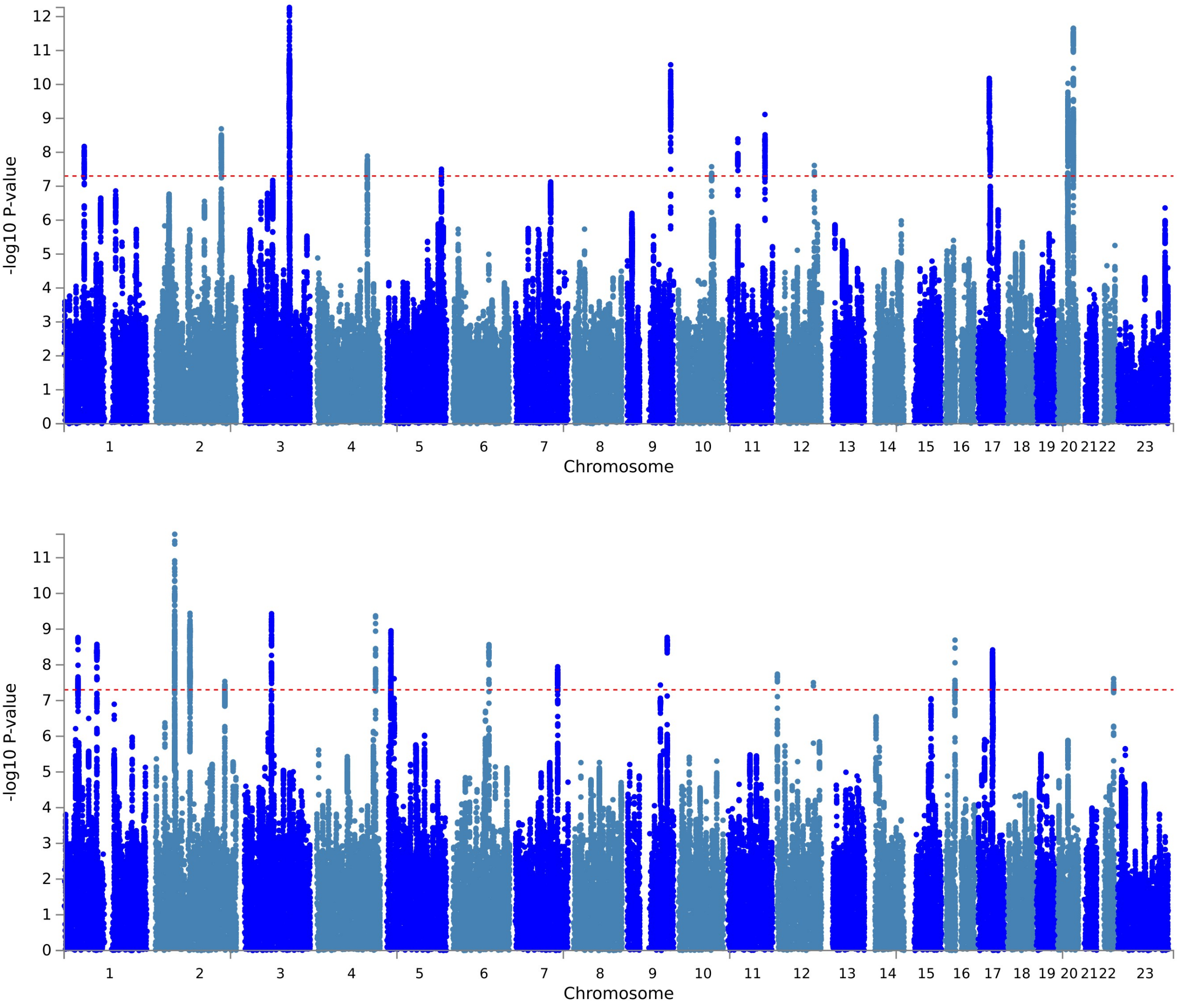

Supplementary Figure 4: Manhattan plots for the independent factors of dyslexia (top) and rhythm impairment (bottom). The bivariate GWAS was performed using Genomic SEM. Each data point represents a SNP. The *y* axes show -log10(P) values, and the *x* axes show genomic coordinates. The red lines correspond to genome-wide significance threshold ($P<5\times10^{-8}$). $N_{rhythm\ impairment}$=606,825, $N_{dyslexia}$=1,138,870.

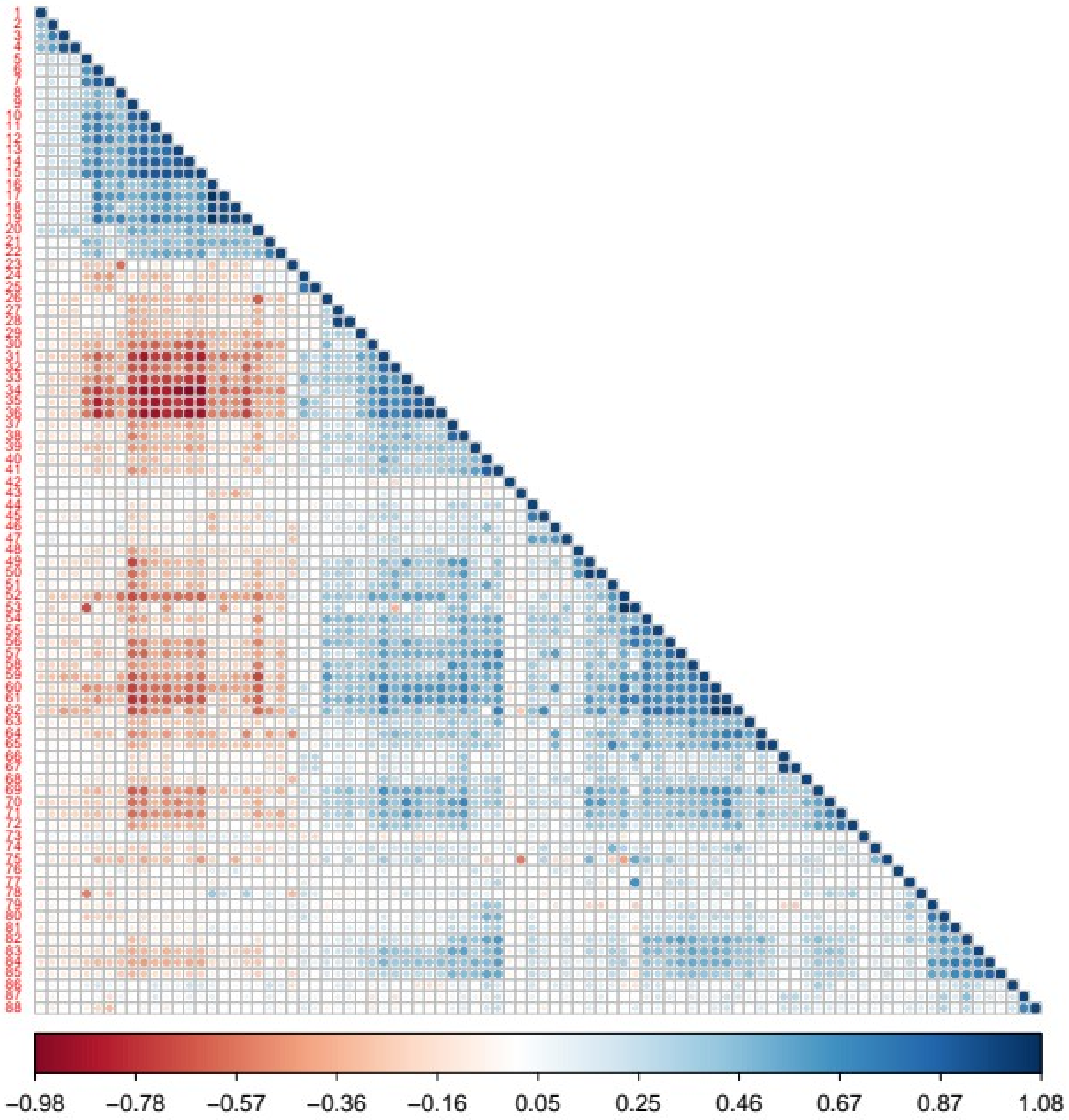

Supplementary Figure 5: The genetic correlation (r_g) matrix among 88 traits that are significantly correlated (P_{FDR}<0.05) with either dyslexia or musical rhythm. Traits were curated from Doust et al. (2022) and Niarchou et al. (2022). Genetic correlations were estimated using LDSC. The colour code in each cell represent the r_g between the intersecting traits. The numbers on the left-hand side correspond to the ID of each trait (see Table S13 for a full list of traits and trait IDs). The colour code bar at the bottom indicates the magnitude and direction of r_g (red: negative correlation; blue: positive correlation; white: no correlation).

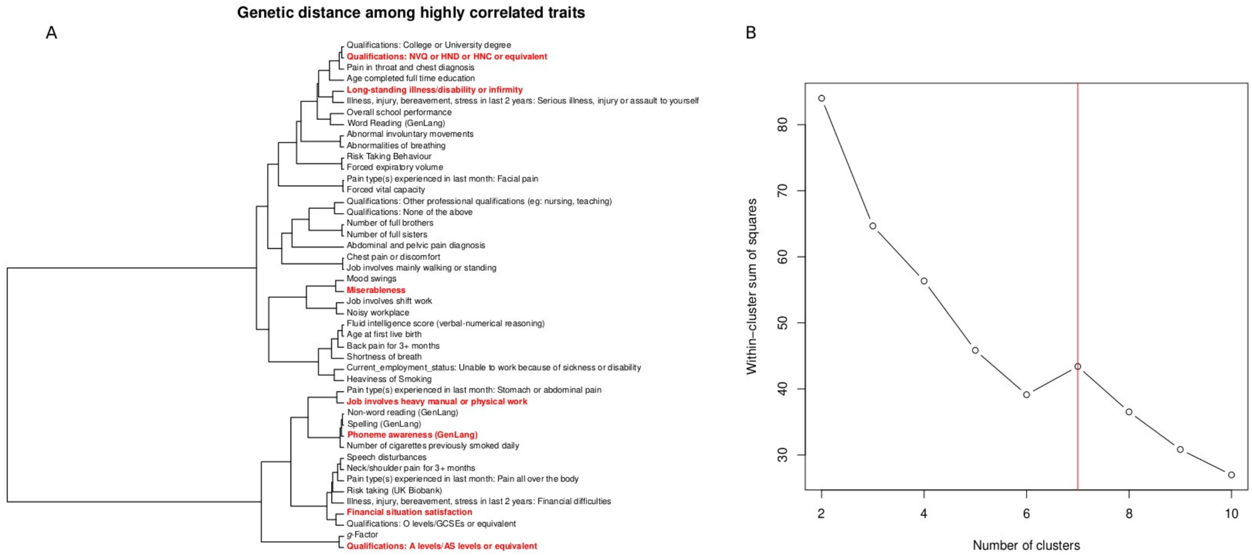

Supplementary Figure 6: (A) Dendrogram showing the hierarchical clustering of highly genetically correlated ($|r_g|$>0.80) traits. (B) Knee-point algorithm plot showing seven representative clusters. For each cluster, one representative trait (shown in bold red) was used in the genetic correlation analysis with the $F_{gRI\text{-}D}$.

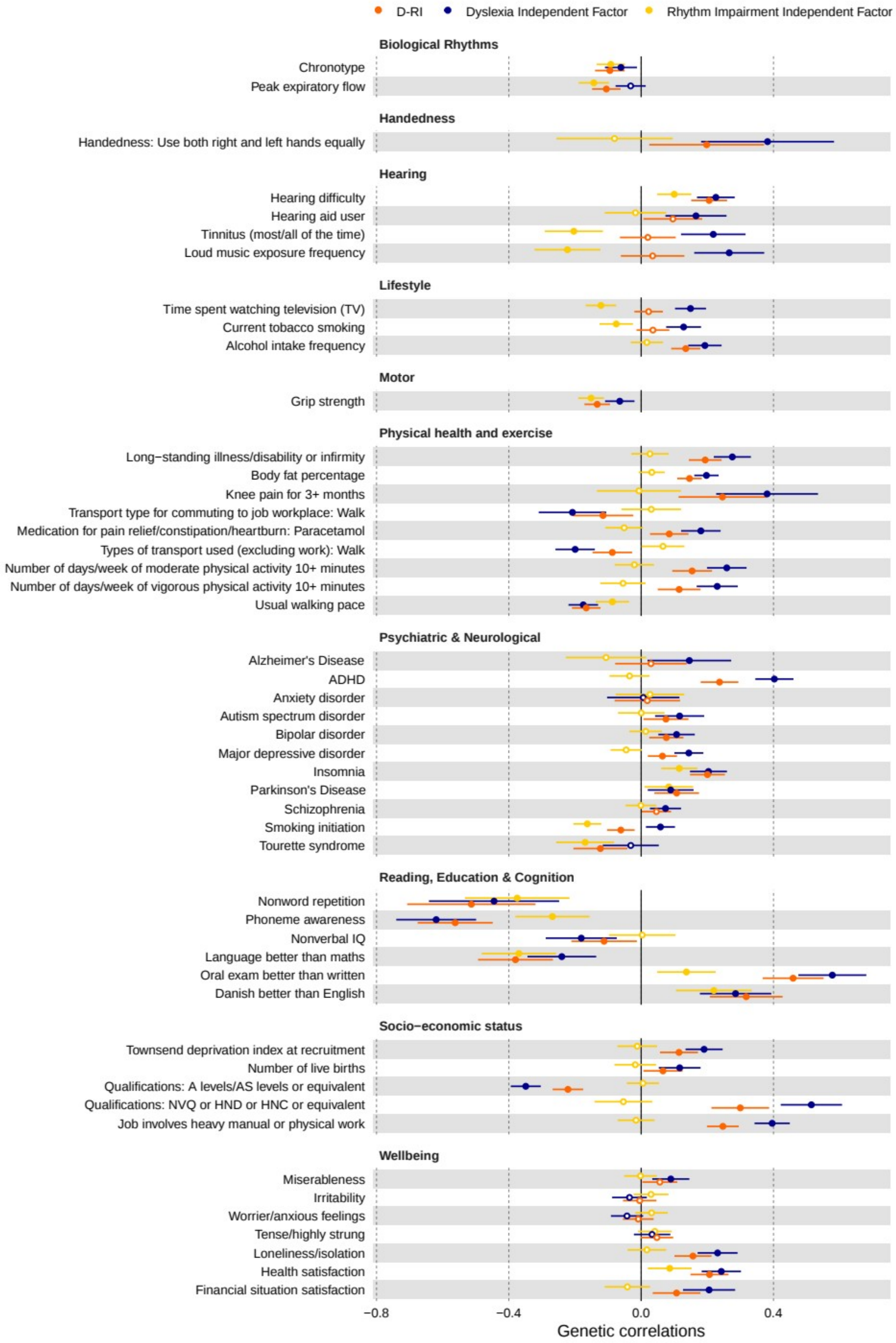

Supplementary Figure 7: Genetic correlations (r_g) between 49 selected traits and $F_{gRI\text{-}D}$ (D-RI) (orange), and dyslexia (blue) and musical rhythm impairment (yellow) independent factors. Genetic correlations were estimated using LDSC. Full circles indicate significant correlations after FDR correction for 49 tests (P_{FDR}<0.05). Error bars represent standard errors of the mean, with whiskers extending one SEM below/above the r_g. The sample size of each trait is shown in Table S13.

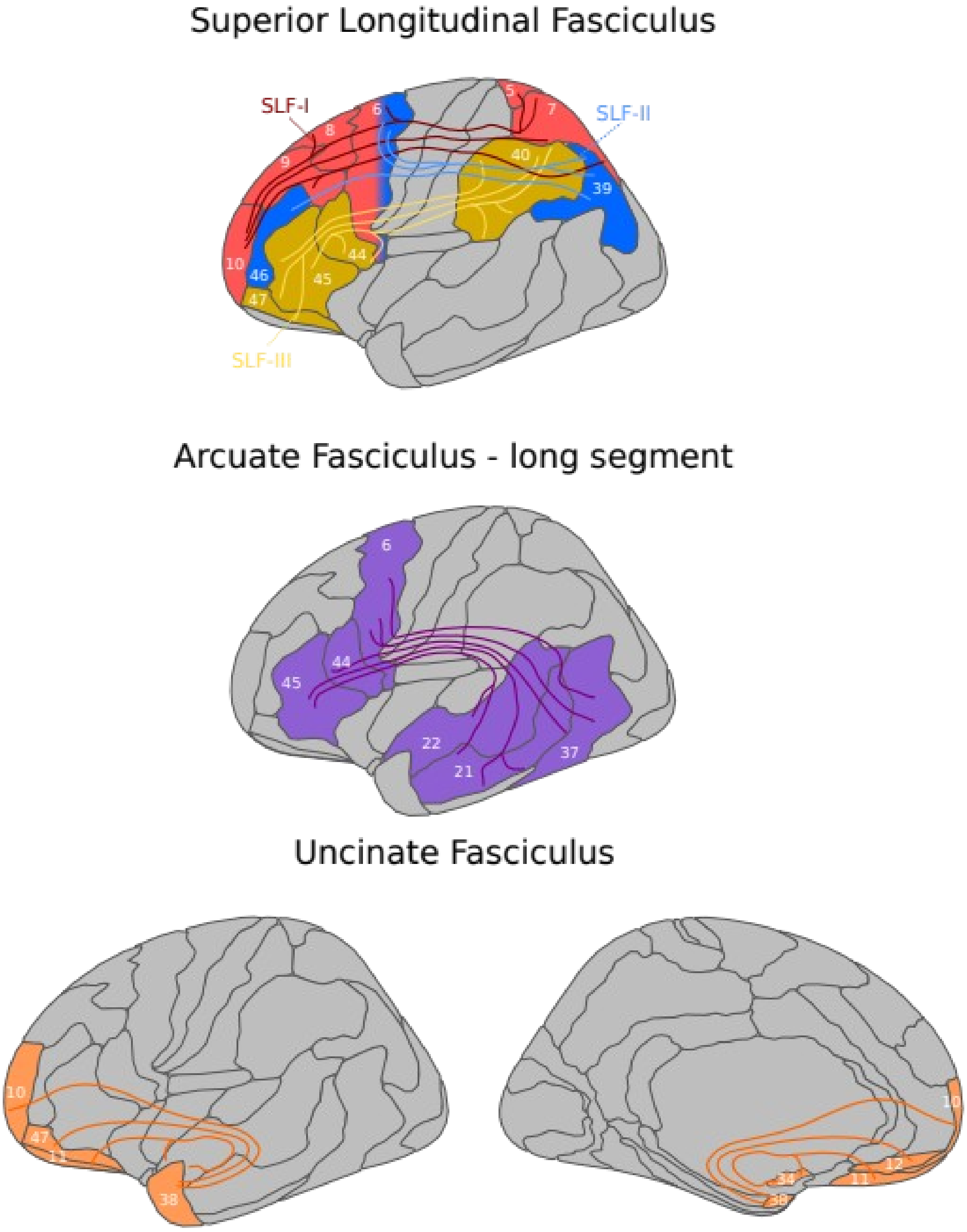

Supplementary Figure 8: Brain plots showing five left hemisphere white-matter tracts investigated in our local genetic correlation analysis with $F_{gRI\text{-}D}$. Note that only the left hemisphere of the brain is shown here from the lateral view for the superior longitudinal fasciculus and the arcuate fasciculus long segment. For the uncinate fasciculus, both lateral (left) and medial (right) views of the left hemisphere are shown. Each white-matter tract has the same colour with the Brodmann Areas that it connects. Numbers correspond to Brodmann Area IDs. Brodmann Area 6 is connected to both SLF-I and SLF-II, thus it is depicted as both red and blue.

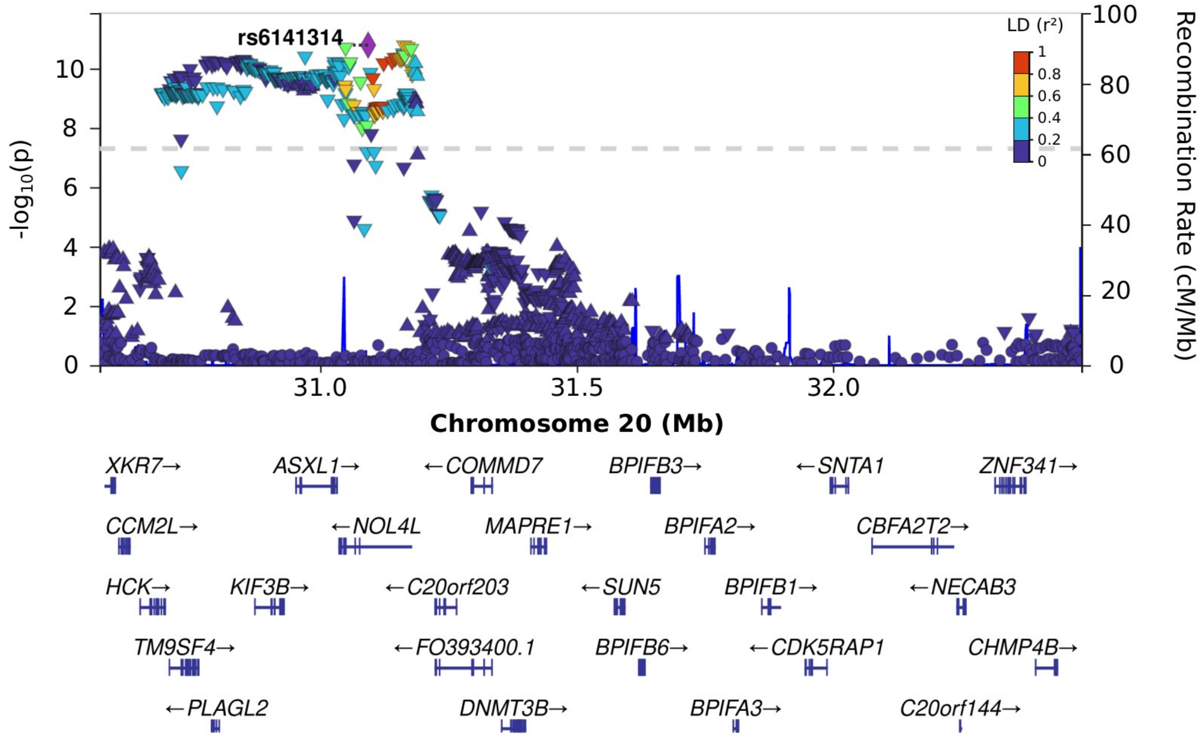

Supplementary Figure 9: LocusZoom plot of the chr20: 30,569,660-32,484,506 locus, the region which is identified by local genetic correlation analysis of $F_{gRI\text{-}D}$ and Superior Longitudinal Fasciculus I. The *x* axis shows genomic locations, and the *y* axis shows $F_{gRI\text{-}D}$ $-\log_{10}$(P)-values on the left-hand side and recombination rates (centiMorgan per megabase) on the right-hand side. Direction of the triangles represent effect directions (upwards: positive; downwards: negative). LD (r^2) levels with rs6141314 are colour-coded. Grey dashed line indicates the genome-wide significance level ($P<5\text{x}10^{-8}$).
