# Supplementary material for: The shared genetic architecture and evolution of human language and musical rhythm
Source: Nat Hum Behav. 2024 Nov 21;9(2):376–90. doi: 10.1038/s41562-024-02051-y (PMC11860242; doi:10.1038/s41562-024-02051-y)
Supplement: Supplementary file 2 — Reporting Summary [file 41562_2024_2051_MOESM2_ESM.pdf]

nature portfolio

Corresponding author(s): Gökberk Alagöz
Simon E. Fisher
Reyna L. Gordon

Last updated by author(s): Sep 17, 2024

Reporting Summary

Nature Portfolio wishes to improve the reproducibility of the work that we publish. This form provides structure for consistency and transparency in reporting. For further information on Nature Portfolio policies, see our Editorial Policies and the Editorial Policy Checklist.

Statistics

For all statistical analyses, confirm that the following items are present in the figure legend, table legend, main text, or Methods section.

n/a	Confirmed	
☐	☒	The exact sample size (n) for each experimental group/condition, given as a discrete number and unit of measurement
☐	☒	A statement on whether measurements were taken from distinct samples or whether the same sample was measured repeatedly
☐	☒	The statistical test(s) used AND whether they are one- or two-sided<br>*Only common tests should be described solely by name; describe more complex techniques in the Methods section.*
☐	☒	A description of all covariates tested
☐	☒	A description of any assumptions or corrections, such as tests of normality and adjustment for multiple comparisons
☐	☒	A full description of the statistical parameters including central tendency (e.g. means) or other basic estimates (e.g. regression coefficient) AND variation (e.g. standard deviation) or associated estimates of uncertainty (e.g. confidence intervals)
☐	☒	For null hypothesis testing, the test statistic (e.g. F, t, r) with confidence intervals, effect sizes, degrees of freedom and P value noted<br>*Give P values as exact values whenever suitable.*
☒	☐	For Bayesian analysis, information on the choice of priors and Markov chain Monte Carlo settings
☒	☐	For hierarchical and complex designs, identification of the appropriate level for tests and full reporting of outcomes
☐	☒	Estimates of effect sizes (e.g. Cohen's d, Pearson's r), indicating how they were calculated

Our web collection on statistics for biologists contains articles on many of the points above.

Software and code

Policy information about availability of computer code

Data collection	23andMe, Inc. and UK Biobank use a custom pipeline for data collection.
Data analysis	R (v4.0.3), LD score regression (v1.0.1), PLINK (v1.9), GenomicSEM (v0.0.5c), GWAMA (v1.2.6), CPASSOC (v1), MAGMA (v1.10), GCTB (v2.02), LAVA (v0.1.0). All scripts used in this study are made publicy available at https://github.com/galagoz/pleiotropyevo. 23andMe, Inc. and the UK Biobank use a custom pipeline for data collection.

For manuscripts utilizing custom algorithms or software that are central to the research but not yet described in published literature, software must be made available to editors and reviewers. We strongly encourage code deposition in a community repository (e.g. GitHub). See the Nature Portfolio guidelines for submitting code & software for further information.

Data

Policy information about availability of data

All manuscripts must include a data availability statement. This statement should provide the following information, where applicable:

- Accession codes, unique identifiers, or web links for publicly available datasets
- A description of any restrictions on data availability
- For clinical datasets or third party data, please ensure that the statement adheres to our policy

The full GWAS summary statistics from the original 23andMe discovery studies set have been made available through 23andMe to qualified researchers under an agreement with 23andMe that protects the privacy of the 23andMe participants. Datasets will be made available at no cost for academic use. Please visit https://

research.23andme.com/collaborate/#dataset-access/ for more information and to apply to access the data. Participants provided informed consent and volunteered to participate in the research online, under a protocol approved by the external AAHRPP-accredited IRB, Ethical & Independent (E&I) Review Services. As of 2022, E&I Review Services is part of Salus IRB (https://www.versiticlinicaltrials.org/salusirb). The primary neuroimaging genetics data used in this study are available via the UK Biobank website www.ukbiobank.ac.uk. The GWAS summary statistics of fractional anisotropy measures of five white-matter tracts, which were derived from the UK Biobank brain imaging data set, are publicly available at the MPI Archive (accession link: https://hdl.handle.net/1839/d99a85d0-537f-46a2-af19-ee5310311ec8). Genome annotation of the human genome assembly (hg19) was downloaded from the NCBI database (https://www.ncbi.nlm.nih.gov/datasets/gene/GCF_000001405.25).

Research involving human participants, their data, or biological material

Policy information about studies with human participants or human data. See also policy information about sex, gender (identity/presentation), and sexual orientation and race, ethnicity and racism.

Reporting on sex and gender	23andMe dyslexia GWAS: Number of cases = 51,800 (21,513 male, 30,287 female), Number of controls = 1,087,070 (446,054 male, 641,016 female).<br>23andMe beat-synchronisation GWAS: Total number of cases = 555,660, Number of controls = 51,165 (59% of the total sample size are females).<br>UK Biobank white-matter connectivity GWAS: Total sample size = 31,465 (14,968 male, 16,497 female).
Reporting on race, ethnicity, or other socially relevant groupings	Our study sample is limited by the European-ancestry individuals to minimise the population stratification and confounding on GWAS effects.
Population characteristics	Population characteristics of dyslexia and beat-synchronisation GWAS study samples are described in the original papers. We used neuroimaging and genotype data from the UK Biobank to perform white-matter connectivity GWASs (N=31,465, mean age=55.21, range between 40 to 70 years old, 16,497 females). The following covariates were used for the white-matter connectivity GWASs: age, sex, genotype array type, assessment centre, and ten genetic principal components capturing population genetic diversity.
Recruitment	Participants are customers of 23andMe, so are invited to participate in general research. There is under-representation of low socio-economic position and all participants are over 18 years. The UK Biobank enrolled participants aged 40-69 between 2006 and 2010 for baseline assessments in 22 centres across the UK. The assessment visits comprised interviews and questionnaires covering lifestyles and health conditions, physical measures, biological samples, imaging, and genotyping.
Ethics oversight	23andMe's human subject protocol was reviewed and approved by Ethical & Independent Review Services, a private institutional review board. UK Biobank has received ethical approval from the North West Multi-centre research Ethics Committee (MREC), and informed consent through electronic signature was obtained from study participants.

Note that full information on the approval of the study protocol must also be provided in the manuscript.

Field-specific reporting

Please select the one below that is the best fit for your research. If you are not sure, read the appropriate sections before making your selection.

☒ Life sciences ☐ Behavioural & social sciences ☐ Ecological, evolutionary & environmental sciences

For a reference copy of the document with all sections, see nature.com/documents/nr-reporting-summary-flat.pdf

Life sciences study design

All studies must disclose on these points even when the disclosure is negative.

Sample size	Sample sizes (beat-synchronisation N=606,825; dyslexia N=1.138.870) were determined by data availability. This range of sample size has been successfully used in many genome-wide association studies of diverse human phenotypes.
Data exclusions	Data exclusion in two original GWAS studies are described in Niarchou et al. (2022) and Doust et al. (2022). For LAVA analysis using diffusion-weighted MRI data, we excluded participants with unusual heterozygosity (>0.19) high missingness (>0.05), sex mismatches between genetically inferred sex and self-reported sex as reported by Bycroft et al. (2018).
Replication	Original dyslexia and musical rhythm GWASs (Doust et al., Niarchou et al.) were not divided into discovery and replication samples to maximise the statistical power for genomic-signal discovery. However, we replicated the multivariate GWAS results from Genomic SEM by using two independent multivariate GWAS tools (GWAMA and CPASSOC), and succesfully replicated the shared genetic loci between dyslexia and musical rhythm impairment identified by Genomic SEM (see Supplementary Figure 3).<br><br>Data accession guidelines and accession number in the Data Availability section, scripts provided in the Code Availability section, and the analysis results provided in the Supplementary Information and Supplementary Tables files should be sufficient for other researchers to attempt replication.
Randomization	Not relevant, this was an observational study.
Blinding	Blinding was not used. Analysts were not blind to phenotypic status.

Reporting for specific materials, systems and methods

We require information from authors about some types of materials, experimental systems and methods used in many studies. Here, indicate whether each material, system or method listed is relevant to your study. If you are not sure if a list item applies to your research, read the appropriate section before selecting a response.

Materials & experimental systems

n/a	Involved in the study	
☒	☐	Antibodies
☒	☐	Eukaryotic cell lines
☒	☐	Palaeontology and archaeology
☒	☐	Animals and other organisms
☒	☐	Clinical data
☒	☐	Dual use research of concern
☒	☐	Plants

Methods

n/a	Involved in the study	
☒	☐	ChIP-seq
☒	☐	Flow cytometry
☐	☒	MRI-based neuroimaging

Plants

Seed stocks: *Report on the source of all seed stocks or other plant material used. If applicable, state the seed stock centre and catalogue number. If plant specimens were collected from the field, describe the collection location, date and sampling procedures.*

Novel plant genotypes: *Describe the methods by which all novel plant genotypes were produced. This includes those generated by transgenic approaches, gene editing, chemical/radiation-based mutagenesis and hybridization. For transgenic lines, describe the transformation method, the number of independent lines analyzed and the generation upon which experiments were performed. For gene-edited lines, describe the editor used, the endogenous sequence targeted for editing, the targeting guide RNA sequence (if applicable) and how the editor was applied.*

Authentication: *Describe any authentication procedures for each seed stock used or novel genotype generated. Describe any experiments used to assess the effect of a mutation and, where applicable, how potential secondary effects (e.g. second site T-DNA insertions, mosiacism, off-target gene editing) were examined.*

Magnetic resonance imaging

Experimental design

Design type: No experimental design

Design specifications: *Specify the number of blocks, trials or experimental units per session and/or subject, and specify the length of each trial or block (if trials are blocked) and interval between trials.*

Behavioral performance measures: *State number and/or type of variables recorded (e.g. correct button press, response time) and what statistics were used to establish that the subjects were performing the task as expected (e.g. mean, range, and/or standard deviation across subjects).*

Acquisition

Imaging type(s): Diffusion-weighted MRI

Field strength: 3T

Sequence & imaging parameters: The UKB Diffusion-weighted MRI images were acquired with five non diffusion-weighted image b = 0 s/mm2, diffusion-weighting of b = 1000, and 2000s/mm2 with 50 directions each. MRI data acquisition details are fully provided here: https://www.nature.com/articles/nn.4393

Area of acquisition: Whole brain

Diffusion MRI: ☒ Used ☐ Not used

Parameters: Please see "Sequence & imaging parameters" section above for full details.

Preprocessing

Preprocessing software: (see https://www.nature.com/articles/nn.4393 and https://www.sciencedirect.com/science/article/pii/S1053811917308613)

Normalization: (see https://www.nature.com/articles/nn.4393 and https://www.sciencedirect.com/science/article/pii/S1053811917308613)

Normalization template	(see https://www.nature.com/articles/nn.4393 and https://www.sciencedirect.com/science/article/pii/S1053811917308613)
Noise and artifact removal	(see https://www.nature.com/articles/nn.4393 and https://www.sciencedirect.com/science/article/pii/S1053811917308613)
Volume censoring	(see https://www.nature.com/articles/nn.4393 and https://www.sciencedirect.com/science/article/pii/S1053811917308613)

Statistical modeling & inference

Model type and settings	We made use of categorical and continuous variables controlling for covariates in the genome-wide association studies including age, sex, genotype array type, and assessment centre. To avoid possible confounding effects related to ancestry, we used the first ten genetic principal components capturing population genetic diversity.
Effect(s) tested	Genetic effects on white-matter connectivity measures, as described in the paper.

Specify type of analysis: ☐ Whole brain ☒ ROI-based ☐ Both

Anatomical location(s)	We averaged the fractional anisotropy skeletonised image across a set of five left hemishere white-matter tract defined from a probabilistic atlas (Rojkova et al. 2016).
Statistic type for inference (See Eklund et al. 2016)	Local Analysis of [co]Variant Association (LAVA; see https://www.nature.com/articles/s41588-022-01017-y)
Correction	FDR

Models & analysis

n/a	Involved in the study
☒	☐ Functional and/or effective connectivity
☒	☐ Graph analysis
☒	☐ Multivariate modeling or predictive analysis
